# Eradication of PRRS from Hungarian Pig Herds between 2014 and 2022

**DOI:** 10.3390/ani13243747

**Published:** 2023-12-05

**Authors:** István Szabó, Imre Nemes, Lajos Bognár, Zsolt Terjék, Tamás Molnár, Tamás Abonyi, Ádám Bálint, Dávid G. Horváth, Gyula Balka

**Affiliations:** 1 National PRRS Eradication Committee, Keleti Károly. u. 24, 1024 Budapest, Hungary; iszabodr@t-online.hu (I.S.); nemesi@nebih.gov.hu (I.N.); zsoltdrterjek@gmail.com (Z.T.); abonyit@nebih.gov.hu (T.A.); 2 Chief Veterinary Officer of Hungary, Ministry of Agriculture, Kossuth Lajos t. 11, 1055 Budapest, Hungary; lajos.bognar@am.gov.hu; 3 Veterinary Diagnostic Directorate, National Food Chain Safety Office, Tábornok u. 2, 1143 Budapest, Hungary; balintad@nebih.gov.hu; 4 Department of Pathology, University of Veterinary Medicine, István u. 2, 1078 Budapest, Hungary; horvath.david.geza@univet.hu; 5 National Laboratory of Infectious Animal Diseases, Antimicrobial Resistance, Veterinary Public Health and Food Chain Safety, University of Veterinary Medicine, 1078 Budapest, Hungary

**Keywords:** pig, PRRS, Hungary, eradication

## Abstract

**Simple Summary:**

The authors’ aim was to summarise the general concept and approach of the Hungarian National PRRS Eradication Program. However, limits on the length of the paper meant that many important points of the process could not be described in full detail. In some cases, previous publications were cited to guide the readers to a more detailed description of particular eradication parts.

**Abstract:**

Porcine reproductive and respiratory syndrome (PRRS) is a widespread infectious disease that is currently a major cause of economic losses in pig production. In Hungary, a National PRRS Eradication Program has been introduced to attain a more efficient, economic, and competitive international market position. The program has been also approved by the EU, but the resulting legal obligations have imposed a burden on Hungarian producers to comply with EU competition rules. The implementation of the program has been carried out by the veterinary authorities with the consent of, continuous support from and monitoring conducted by organisations within the pig sector as well as a scientific committee. The PRRS eradication program in Hungary was based on a regional territorial principle and was compulsory for all pig holdings within the regions. In Hungary, large fattening farms operate as all-in/all-out or continuous flow systems. Large-scale breeding herds are predominantly farrow-to-finish types. Although its significance has decreased in recent decades, 20% of the Hungarian pig population is still kept on small (backyard) farms (<100 animals). All PRRSV-infected large-scale farms had to develop a unit-adapted eradication plan, including external and internal biosecurity measures, vaccinations, etc. It was crucial to render each fattening unit free of the disease, as fattening units play a significant role in spreading the virus within the country. The eradication efforts mainly implemented were depopulation–repopulation methods, but on some farms a testing and removal method has been used. As the eradication progressed over the years, the introduction of infected fattening pigs was restricted. Thanks to these measures, Hungarian large-scale fattening farms became PRRSV-free by the end of 2018. The PRRSV-free status of small-scale herds was achieved by the end of 2015 and was maintained between 2016 and 2021. By 31 December 2021, all breeding pigs in large-scale farms in Hungary were free of wild-type PRRS virus. By 31 March 2022, the total pig population of the country, including all backyard farms and fattening units, achieved PRRSV-free status. The future goal is to ensure and maintain the PRRSV-free status of Hungary via strict import regulations of live animals combined with the continuous and thorough screening of incoming and resident herds for the presence of the virus.

## 1. Introduction

Pig breeding in Hungary has been characterised for many decades by the parallel presence of two different forms of farming. Large-scale pig farms produce pigs for the market, while individual farmers (backyard farms) keep one or two sows and a limited number of pigs for fattening (1–10) for on-the-spot processing and consumption. Even before Hungary’s accession to the European Union, the market role of backyard farms had declined significantly. In 1997, their market share was 53.7%, in 2004 (i.e., in the year of Hungary’s accession to the EU) it was 41.6%, and in 2019 only 18.4% of pigs were kept in backyard farms [1].

In Hungary, the average herd size of large-scale pig holdings (2933 pigs/herd in 2016) is exceptionally high compared to other EU countries, being the second highest after Denmark (3182 pigs/herd) [2].

**Figure 1:**
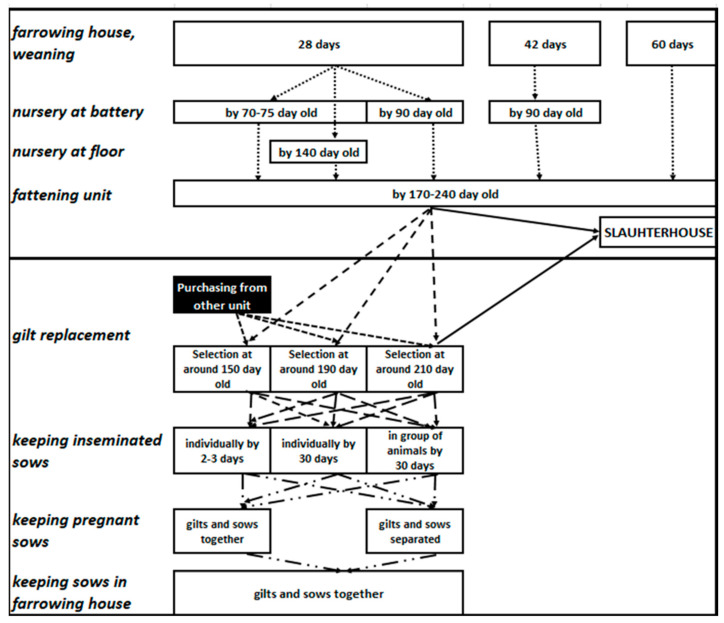
Variability of the technological processes in large-scale pig farms in Hungary.

Large-scale pig farms of Hungary are predominantly (>85%) farrow-to-finish, resulting in the concentration of reproduction, farrowing, pre-fattening, fattening and replacement gilt units on the same farm. In the vast majority of Hungarian farms, only breeding boars or semen are imported from external sources. In Hungarian large-scale pig farms, there are no uniform housing, feeding and disease control technologies and no preventive or therapeutic treatment protocols (Figure 1).

The majority of large-scale industrialised and specialised swine units were built in the 1970s according to the technological levels of that period. Since their construction, the amount of money spent on renovation or complete reconstruction of pig farms has been inadequate. More modern farms were built on the principle of multi-site production, where the different stages of pig production (farrowing, nursery, fattening) take place on different sites. This design is significantly better than farrow-to-finish systems in terms of infectious disease control [3].

Traditionally, fattening pig production in Hungary takes three different forms.

I. The majority of slaughtered pigs are produced in farrow-to-finish pig farms.
II. In recent years, large-scale breeding herds have emerged where only weaned piglets or pre-fatteners are reared at the breeding establishments, and either pre-fattening or fattening is carried out at other locations often run by different owners (multi-site system). In the case of large-scale fattening farms (with a minimum of 100 pigs/holding), the animals may come from the same holding, from other herds in Hungary
III. or from abroad [4].

Large-scale fattening farms can operate in two ways: all-in/all-out systems or continuous operation sites [3,4,5].

According to slaughterhouse data, the average slaughter live weight of a pig is 104–118 kg and carcass weight is 83–95 kg. In Hungary, per capita pork consumption significantly declined in the 1990s, and since the 2000s, pork and poultry meat have accounted for 90% of the total meat consumption in equal proportions. The average domestic pork consumption was 38.8 kg per capita in 1990 and 29.1 kg per capita in 2020 [6].

Porcine reproductive and respiratory syndrome (PRRS) is one of the most important infectious diseases of pigs, causing the highest economic damage to the pork industry worldwide [7,8]. According to a study conducted in the Midwest US, the average PRRS outbreak reduced production by about 7.4% compared to disease-free periods, and it resulted in 1.92 fewer piglets per sow [9]. In the USA, PRRS causes a loss of $114.71 per sow and $4.67 per slaughter pig [10]. In Scotland, PRRS costs in the pig sector are £80 per sow and £3.5 per slaughter pig [11,12]. PRRS even causes significantly more damage to the global pig industry than African swine fever [13]. Based on the study of 21 farms in Germany, Renken et al. [14] calculated the loss caused by PRRS at €255 per sow (HUF ~96,000/sow) per year.

Accordingly, in Hungary, with 4 million slaughtered pigs and 170,000 breeding sows [15], the estimated loss due to PRRSV infection is close to HUF 5 billion (≈€14 m) per year.

The Pork and Food Chain Safety Strategy of the Hungarian Government (2013–2022) highlights the need to eradicate the PRRS virus (PRRSV). The implementation of the strategy emphasises the importance of the pork industry for the Hungarian agricultural economy, despite the significant decline in the swine population over the past years. Following the successful implementation of the Aujeszky’s disease eradication program [16], the elimination of PRRSV from Hungarian swine populations would help to increase the potential of the pig meat market.

The legal background of the eradication program was laid down in Decree 3/2014 (16 January, [17]) by the Minister of Agriculture. The competent committee of the European Union approved the program with state participation. According to the principles of the eradication program, the National PRRS Eradication Plan specifies the measures to be taken against PRRSV and defines the executives of the program. In order to ensure a uniform implementation of the plan, on the 18 February 2014 the Chief Veterinary Officer of Hungary appointed a National PRRS Eradication Committee to address professional, epidemiological, administrative or other issues arising during the process. The officials (Ministry of Agriculture, Ministry, and National Food Chain Safety Office (NÉBIH)) held discussions with the representative bodies of domestic pig farmers during the eradication planning period. The National PRRS Eradication Committee (NEC) invited all pharmaceutical companies with a registered PRRS vaccine in Hungary to share their own control/eradication program, in addition to assisting in the implementation of the program.

The administrative territory of Hungary is divided into seven regions based on the Nomenclature of Territorial Units for Statistics (NUTS) of the European Union. These seven regions include nineteen counties and one additional unit, Budapest (the capital of Hungary).

The implementation of the PRRS eradication program for pigs in Hungary was based on the territorial principle. This meant that PRRS had to be eradicated from the entire pig population of a given administrative unit (district, county and region) within a specified time period [18,19,20,21,22,23,24]. This was the only approach that was highly likely to avoid the infection or re-infection of PRRSV-free pig herds.

## 2. Materials and Methods

The eradication of a specified infectious disease from an animal species at a national level consists of three phases: planning, organising and implementing the eradication campaign [25]. The most important element of the planning process is to have up-to-date information on the animal health status of the country’s livestock. 

In the PRRS eradication process, the ‘preparation and planning’ phase involved identifying the location of PRRSV-infected (as well as PRRSV-free) swine herds in the country. This method required the establishment of strict qualification rules that clearly defined which pigs were considered to be (i) infected, (ii) suspected of being infected or (iii) free from PRRSV. These criteria made it necessary to establish clear requirements for the classification of swine farms with regard to PRRS. Decree 3/2014 (16 January) of the Ministry of Agriculture [15] provided the basis for this work. Every governmental action was based on the advice of the National PRRS Eradication Committee. The main principle of the decree is that all large-scale pig farms and all establishments with pigs have to be certified by the district veterinary authority for PRRS status. The Decree specifies that a herd is considered free from PRRSV according to the following:

(a) proper conditions of disease control (biosecurity) measures are available in the herd;
(b) the herd has been subjected to annual qualification in accordance with the regulation, and has been found free from infected animals;
(c) in the case of breeding herds, the sows or gilts have been bred with a confirmed negative boar or have been inseminated with semen from such males.

Taking into account the principle of territorial eradication, during the ‘organisation’ phase (2014–2016), version 1.0 of the National PRRS Eradication Plan was prepared and published as directed by the Chief Veterinary Officer of Hungary.

**Figure 2:**
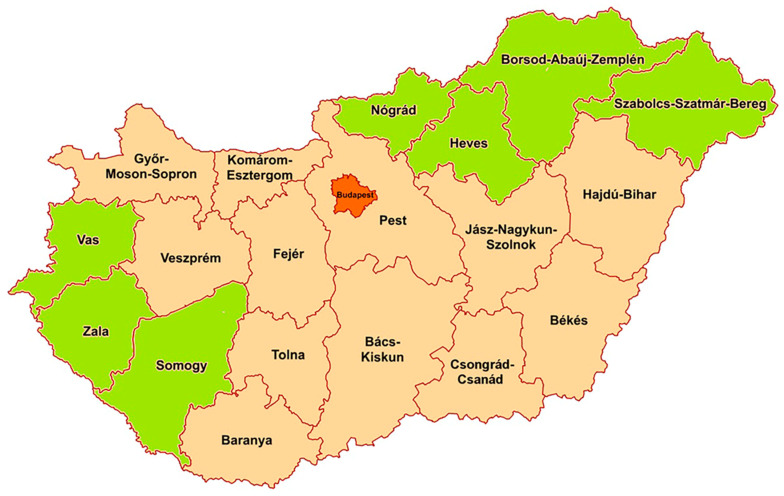
Counties marked in green defined as ‘areas under eradication processes’ at the beginning of the eradication.

According to the regulation, the plan identified the counties that were classified as ‘areas under eradication processes’. These areas were defined by the National Food Chain Safety Authority where eradication processes were implemented under the provisions of the Departmental Government Office for Food Chain Safety and Animal Health. These areas included the whole region of Northern Hungary, i.e., Nógrád, Heves, Borsod-Abaúj-Zemplén (BAZ) and Szabolcs-Szatmár-Bereg counties, as well as Vas, Zala and Somogy counties (Figure 2).

The National PRRS Eradication Plan required the establishment of separate PRRS eradication plans for each large-scale pig holding, including large farrow-to-finish farms. The eradication plan was approved by the competent county veterinary authority on 31 May 2015 [3,4,5].

From 2014 onwards, a laboratory serological confirmation regime was carried out every year as specified by the Decree. The required tests were carried out using funding from the state. Based on the results of the examinations, the herds were officially qualified by the veterinary authority. 

If a pig farm was identified as infected with PRRSV, several steps were taken depending on the type of holding:

(a) Backyard farms: the authority ordered the depopulation of the herds with state compensation, but without the need for repopulation.
(b) Large-scale fattening only units: at this early stage of eradication, after the pigs were transported to slaughter, the premises had to be thoroughly cleaned and disinfected, and only PRRSV-free animals could be used for repopulation.
(c) Large-scale breeding farms: complete freedom to decide whether to eradicate the PRRS virus via complete depopulation–repopulation or other methods (herd closure, testing and removal, etc.). The goal was to find the optimal eradication method tailored to each farm based on its technology, production system and management.

The eradication plans have been developed by the management of the various swine herds assisted by the PRRS Expert Veterinary Team delegated by the Chief Veterinary Officer of Hungary and by the technical specialists representing the pharmaceutical companies selling PRRS vaccines in Hungary. The professional evaluation and approval of every eradication plan was carried out by the National PRRS Eradication Committee following a request for official approval by the veterinary authority.

At its 85th General Meeting in May 2017, the World Organisation for Animal Health (WOAH, formerly OIE) approved the international PRRS regulations in the Terrestrial Animal Health Code [26]. This regulation was more permissive for animals/herds with proven infection with the PRRSV MLV strain only. 

Because of this interpretation, the decision of the Chief Veterinary Officer of Hungary allowed the introduction of the concept and category of PRRS ‘vaccinated free’ (VF) swine herds. This term applies to all three types of breeding in large-scale swine farms (farrowing to finish, farrowing to pre-fattening (nursery) and farrowing to weaning). In principle, breeding stocks can be immunised without time limitation, but the PRRSV-free status of the offspring of any age group must be confirmed via laboratory methods (ELISA and PCR). Vaccination is not allowed during the growing phase (including lactation, nursery, fattening) in these farms. Therefore, the tests must not show seropositivity at the end of the fattening period of the offspring, and all animals must be PRRSV-free, including even the vaccine virus.

Since the beginning of the eradication program, an ELISA method (INgezim, Ingenasa, Spain) has been used to detect antibodies against PRRSV according to the manufacturer’s recommendations. Seropositivity was confirmed or excluded via another confirmatory serological test, indirect immunofluorescence (IIF, [27]). 

For direct molecular PRRSV detection, the Virotype PRRSV RT-PCR Kit (Qiagen, Hilden, Germany) was used according to the manufacturer’s instructions. In case of a positive PCR test, sequencing of the PRRSV ORF5 gene was also attempted. Sequencing was performed by the Sanger method using a BigDye 3.1 kit (Applied Biosystems, Foster City, CA, USA) on an ABI 3500 sequencer (Applied Biosystems). Chromatograms were analysed and edited manually using the BioEdit software version 7.2, available at https://bioedit.software.informer.com/7.2/ (accessed on 28 November 2023) [28]. Based on the sequence data, the ‘similarity network’ image was performed [29] and the sequences were classified as described by Shi et al. [30] and Balka et al. [31]. For the similarity network analysis, a total of 2966 PRRSV ORF5 sequences were identified in Hungary from 2003 onwards. Based on the similarity network, we determined the most probable source of the introduction of PRRSV into a given infected swine herd on a case-by-case basis [29]. 

The second, ‘Eradication Phase’ of the process began based on the decision of the National Chief Veterinary Officer published in November 2017. The decision banned the importation of non-PRRSV-free nursery piglets into Hungary for fattening. In addition, by 30 June 2018, all Hungarian large-scale fattening herds had to be certified PRRSV-free, except those purchasing pre-fatteners from still-infected Hungarian large-scale breeding farms. 

From the second half of 2018, in view of the need to eradicate the disease from large breeding establishments, the PRRS status of large-scale fattening farms was established, with special attention paid to those farms that had not attained a PRRSV-free status (‘Freedom Maintenance Phase’). Farms that used pre-fatteners from Hungarian PRRSV-infected large breeding establishments had to apply disease control measures to ensure that the herd did not pose a risk of infection to non-infected herds in their environment [5].

In 2017, as part of the ‘Eradication Phase’, based on the decision of the National Chief Veterinary Officer (2017):

(a) only pigs from PRRSV free farms could be used for fattening in Hungary (see above for temporary exceptions);
(b) if any diagnostic test showed even one positive result after 48 h of arrival or after 60 days of quarantine, a second test had to be performed. If the second test gave a positive result, the stock had to be sold for slaughter within 15 days or moved outside Hungary, so that the infected stock would not compromise the PRRS status of that area;
(c) from 2019, the PRRSV-free status of the herd of origin had to be certified by the local veterinary authority (regardless of the country of origin).

The eradication of PRRS in independent, large-scale fattening-only herds varied depending on the technology used. Where the all-in/all-out system was used, the infected stock was slaughtered and replaced with a PRRSV-free stock. In farms using continuous production, the infected herd was periodically sold without repopulating until the last delivery. After cleaning and disinfection, repopulation was conducted with PRRSV-free animals.

## 3. Results

### 3.1. Backyard Farms

Between 2012 and 2013, during the ‘Surveillance Phase’, pigs were kept in almost 60% of the settlements in Hungary. Nearly 4% of the backyards surveyed were found to be PRRSV-seropositive in both years. Between 2012 and 2015 sows and some of their offspring on every registered backyard farm were blood tested. Altogether 15–30,000 samples per year were analysed.

The seropositive animals were culled following an official procedure. As a result, on 31 December 2015, small-scale Hungarian swine herds were declared PRRSV-free. The PRRSV-free status was subsequently confirmed via annual PRRS monitoring programs carried out in small-scale swine herds between 2016 and 2021 (Table 1).

We confirmed the presence of the virus via direct virological tests in the seropositive animals. None of the samples from breeding sows were PCR positive. PRRSV PCR positive cases in small-scale herds were typically identified when irregular animal transport, without veterinary authorization, was carried out, or when infected nursery pigs were placed into backyard farms for fattening. This scenario was confirmed via phylogenetic analysis. This scenario was confirmed via ORF5 sequence analysis and the sequences were epidemiologically classified via the similarity network.

Tonsil samples collected from previously seropositive sows at slaughterhouses were uniformly PCR negative for PRRSV.

### 3.2. Large-Scale Breeding Farms

At the beginning of 2014, 345 of 470 large-scale breeding farms were classified as PRRSV free (73%) and 125 (27%) as PRRSV infected. The total number of sows on the 470 farms was 186,404, of which 68,226 (37%) were kept on infected farms. The average number of sows in the herds was 396, 343 in non-infected farms and 546 in infected ones. Except for 20 units, every farm was a farrow-to-finish type.

During the period of the eradication process (2014–2021), 40 large-scale PRRSV-free breeding farms became infected; thus, in total, 165 large-scale breeding farms had to implement their own eradication plan. Out of these 165 farms, 95 were PRRS-eradicated by a depopulation–repopulation method (with 71,011 sows), 5 farms achieved Vaccinated Free (VF) status, several others were found to be seronegative during the certification process, other farms turned from breeding farms to producing fatteners, or some farms closed their premises (Table 2). 

Using ORF5 sequence-based similarity network analysis, we were able to identify the source of infection on all of the above-mentioned 40 farms that were infected sometime between 2014 and 2022. 16 of them were infected by imported nursery pigs. In nearly 50% of the cases, the source of the infection was contaminated vehicles: either the truck transporting the pigs to the slaughterhouse or the one used for the removal of dead animals (Table 3).

By July of 2022, 98.9% of breeding farms in Hungary (which contained 97.7% of large-scale breeding sows) were free of PRRSV. Five farms with their offspring (1.2% in total, which contained 2.3% of the sows kept in large-scale breeding farms) (still vaccinated sows and gilts) were free of the wild-type PRRSV.

### 3.3. Large-Scale Fattening Farms

In 2015, 188 (61.2%) of 307 large-scale fattening farms registered by the Hungarian veterinary authorities had PRRSV-positive animals. In 2013, 46% of the fattened pigs were imported from the Netherlands, 39% from Germany, and 12% from Slovakia. The others came from Denmark, Austria, the Czech Republic, and Slovenia. In 2016, 46% of imported fattened pigs came from the Netherlands, 33% from Germany, 10% from Denmark and 6% from Slovakia [4]. PRRS eradication with the new import rules induced major changes for countries exporting pigs to Hungary: pre-fatteners/nursery pigs could arrive into Hungary only with an official PRRSV-free status certificate. After 2020, pig import from the Netherlands practically ceased because it could not fulfil this requirement; however, imports from Germany, Denmark, the Czech Republic and Slovakia were increased to 53%, 26%, 14% and 5%, respectively [4].

By the end of 2021, all large-scale fattening swine herds in Hungary were PRRSV-free. Based on the authors’ experience, the biggest threat to maintaining Hungarian farms’ PRRSV-free status is the poorly controlled import of nursery pigs. The authors are committed to the effectiveness of the rules implemented from 2017 and consider that is particularly important to request official certificates to prove the PRRSV-free status of nursery pig herds. The importance of this method can be illustrated through the pigs imported from Denmark. Danish trucks (internal transport) transported the pigs from Danish breeding establishments to assembly centres where the animals were picked up by another truck and transported to Hungary. In many cases, during their stay at the assembly centres, pigs coming from both PRRSV-infected and PRRSV-free herds were kept in the same air space, allowing the possibility of infection, despite being reared in PRRSV-free establishments [4].

As a consequence of these events, the largest Hungarian pig importer decided to continue importing nursery animals only if their Danish partner accepts the laboratory test results of the Hungarian National Reference Laboratory (NRL) for PRRSV. The animals are tested for PRRSV (PCR and ELISA) within 48 h of arrival, and if either test is positive, the animals are transported from the farm directly to the slaughterhouse or for fattening outside of Hungary at the exporter’s expense. Following the implementation of these rules, the number of imported Danish fattening pigs decreased dramatically, while in 2020, 156,401 pigs were imported from Denmark, representing 26% of total imports, and in 2022, only 29,325, representing only 4.6% of imports. 

## 4. Discussion

Following the successful eradication of Aujeszky’s disease in 2006, the eradication of PRRS—the infectious pig disease responsible for the most severe economic losses worldwide [7,8,9,10,11,12,13,14]—is a great opportunity for the Hungarian pig sector to improve its market position. 

The Hungarian agricultural leaders identified several goals for the pig sector: to mitigate the economic damage (HUF 5 billion, €13–14 million/year) resulting from PRRSV infection by eradicating of PRRS virus from the Hungarian pig population, to reduce the use of antibiotics, and to achieve long-term sustainable growth with increasing international competitiveness. 

The structure of Hungarian pig production is characterised by the parallel presence of large-scale units of fattening and breeding animals produced for the market as well as backyard farms which keep pigs mainly for family consumption or to supplement family income. In recent years, large-scale fattening farms have been using both an all-in/all-out system at the farm level and a continuous production with all-in/all-out system at the building level. The large-scale breeding farms are predominantly farrow-to-finish. These farms do not have uniform housing, breeding or feeding technologies. The modernization of each production unit and the production method used was not mentioned in the planning phase of national programs as a crucial aspect, even for the eradication of infectious diseases [25].

The eradication of PRRSV from the pig population in Hungary started in 2014 on a territorial principle and was gradually extended to all areas of the country [17]. One of the cornerstones of this approach was the theory that PRRSV can spread up to 9.1 km through the air [32,33]. Based on this and considering that Hungary is a transit country for pigs, the success of the eradication program seemed rather dubious.

However, it was clear to decision makers that achieving a PRRSV-free status could bring significant economic benefits to Hungarian pig production [10,14]. Experts supporting PRRS eradication stressed that successful eradication requires knowledge of the spread of the disease in Hungary, especially under the unique Hungarian swine-keeping circumstances, and this knowledge can be used to develop a proper eradication strategy.

The experts also highlighted that the eradication of Aujeszky’s disease already proved that the infection of backyard farms does not pose a threat to large-scale herds and that the spread of a pathogenic virus from animal to animal is slower (due to the significantly lower number of swine) than in the case of concentrated livestock farms [34].

The successful implementation of PRRS eradication requires the prior assessment of technological diversity among farms; only a program adapted to the specific technology of a given farm can be prosperous. Therefore, each farm required a unique, locally adapted elimination protocol.

Most large-scale swine units became PRRS virus-free by the complete depopulation–repopulation method [18,22,35]. We also found that, although this method has the highest costs, it was the safest way to replace stocks in the shortest possible time. It also enabled the necessary technological renovations and building conservation works to be carried out in the meantime. By implementing this method, it was also possible to switch to modern genetics for a more profitable production. The authorities provided considerable financial support to those who adopted this method. Producers choosing to depopulate and repopulate to eradicate PRRS were entitled to state compensation which was the margin between the breeding value of the sows and their slaughter value (unless there was a reason for not compensating the farmers, e.g., biosecurity or disease control deficiencies).

In Hungary, a slightly higher proportion of farms that used vaccination during PRRS eradication used live vaccines (61%) than inactivated ones (39%) [36,37,38]. Our experience showed that the importance of the basic and farm-specific disease control measures and consistent monitoring of the results via laboratory testing was far more important than the actual type of vaccine used in the given farms. 

The new WOAH (OIE) [26] regulation on PRRS provides a favourable opportunity to achieve a PRRSV-free status in a relatively short time. The new regulation excluded the vaccinated or definitely vaccinated virus-infected animals from the definition of the infected swine and led to the creation of the concept of the “Free Vaccinated” herd. However, in the long term, the process of achieving complete (non-vaccinated and eradicated) breeding holdings for an “MV” status still needs to be developed. In this regard, it is important to remove the previously infected breeding sows as soon as possible and to replace them with PRRSV-free gilts. Our experience showed that the only effective way of PRRS eradication for large numbers of fattening pigs in Hungary is complete depopulation–repopulation.

Maintaining the PRRSV-free status for large-scale pig fattening units at the national level has significantly reduced the long-term cost factor in the Hungarian pig production [12] in conjunction with a significant reduction in antibiotic consumption. The results of the national PRRS eradication program in Hungarian field conditions, using herd depopulation–repopulation with breeding animals of new genetic and animal health status to reach PRRSV-free status, have significantly improved the productivity of the Hungarian pig sector both at individual farm and national level [39].

## 5. Conclusions

The principle of the National PRRS Eradication Program is the specification of a compulsory herd qualification system for all pig farmers, regardless of the number of animals kept. Regular laboratory monitoring of swine herds plays a key role in the Hungarian PRRS eradication process. The continuous maintenance of PRRSV-free status and the external biosecurity and reliable free status of the new replacement herds should be highlighted. This has been greatly facilitated by the repopulation of the fattening stock in Hungary exclusively from PRRSV-free stocks since November 2017.

## Figures and Tables

**Table 1:** Seropositivity rate of backyard farms in Hungary between 2012 and 2021.

Year	The Percentage of PRRSV Seropositivity in Investigated Backyard Farm Pigs
2012	4.68%
2013	3.90%
2014	3.79%
2015	4.33%
2016	1.03%
2017	0.96%
2018	1.12%
2019	1.43%
2020	0.64%
2021	0.15%
2022	0.21%

**Table 2:** PRRS eradication of large-scale swine breeding farms in Hungary between 2014 and 2022.

	Infected Large-Scale Breeding Farms	Sows on the Infected Farms	Average Number of Sows/Farm
2014 basic year	125	68,226	546
Farms that became infected during the national eradication program (new cases) (2014–2022)	40	29,927	748
**Eradication Process**	**Breeding Farms** (**Total**)	**Sows** (**Total**)	**Average Number of Sows/Farm**
Depopulation–repopulation (total)	95	71,011	747
Vaccinated free status	5	3942	788
Other (closed, found to be free, turned to fattening, etc.)	65	23,200	357
Total in Hungary	165	98,153	595

**Table 3:** Farms that became infected between 2014 and 2022 in Hungary.

Total Number of Farms		40	
Types of the farms	farrow to finish	29	73%
	farrow to wean	2	5%
	farrow to nursery	4	10%
	farrow-to-nursery + separated fattening	3	8%
	farrow-to-nursery + some fattening	2	5%
Number of sows	less than 100	8	20%
	101–500	8	20%
	501–1000	13	33%
	above 1001	11	28%
Origin of infection	nursery pigs imported from abroad	16	40%
	virus already present in Hungary before 2014	24	60%
Mode of infection	truck transporting the pigs to the slaughterhouse	10	25%
	truck used to remove dead animals	9	23%
	breeding and fattening farms in the proximity where imported infected nursery pigs were placed	7	18%
	irregular movement of personnel	6	15%
	sperm	2	5%
	possible airborne route	2	5%
	two farms sharing a common manure lake	1	3%
	source unidentified, but foreign virus	2	5%
	only seropositivity was detected, no direct virus detection	1	3%

## Data Availability

The datasets used and/or analysed during the current study are available from the corresponding author upon reasonable request.
